# Carbohydrate mediated drug delivery: Synthesis and characterization of new lipid-conjugates

**DOI:** 10.1016/j.dib.2015.05.026

**Published:** 2015-06-17

**Authors:** Moghis U. Ahmad, Shoukath M. Ali, Ateeq Ahmad, Saifuddin Sheikh, Paul Chen, Imran Ahmad

**Affiliations:** a Jina Pharmaceuticals, Inc., 28100 N. Ashley Circle, Suite 103, Libertyville, IL 60048, USA; b Nia Life Sciences, 28100 N. Ashley Circle, Suite 102, Libertyville, IL 60048, USA

## Abstract

A new synthetic methodology for cationic glycolipids using *p*-aminophenyl-α-d-mannopyranoside (PAPM), *p*-aminophenyl-α-d-galactopyranoside (PAPG) was developed. PAPM-lipids and PAPG-lipids conjugates were also synthesized for targeting drugs to receptors. A binding inhibition study of synthesized *p*-(dimethylamino butylamido) phenyl-α-d-mannopyranoside (**1a)** with Concanavalin A was performed using invertase enzyme. In addition, transfection of pSV-β-gal reporter gene with was investigated in A549 cells.

**Table:** 

Specifications table	
Subject area	Chemistry, lipids, and biology
More specific subject area	Synthesis of carbohydrate-lipid conjugates and its biological activity
Type of data	Synthesis, spectroscopy data, graph, figure
How data was acquired	NMR (Varian Inova 500 MHz), Mass spectroscopy (Nicolet Nexus 470 FT-IR), Optical rotation (Perkin Elmer Polarimeter), IR (Nicolet Nexus 470 FT-IR), UV (SpectraMax M2e, Molecular Devices), cell culture
Data format	Analyzed
Experimental factors	Cells were grown in the Petri Dishes before treatment of samples
Experimental features	Compounds 1a, 2a, 3a, and 1b, 2b, and 3b were synthesized starting from p-nitrophenyl-α-d-mannopyranoside and p-nitrophenyl-α-d-galactopyranoside, respectively. All these compounds were characterized by NMR, IR and Mass spectroscopy, optical rotation etc. Compound 1a was taken as a representative sample for binding-inhibition studies and transfection.
Data source location	NA
Data accessibility	The data is included in this article.

Value of the data

• The article describes the synthesis of new lipid-carbohydrate conjugates.
• The synthesized compounds can be potentially used to target mannose cell surface receptors.
• The synthesized compounds **1a** and **1b** can potentially be used in transfection.

## 1 Experimental design, materials and methods

### 1.1 Synthesis and characterization

**Fig. 1:**
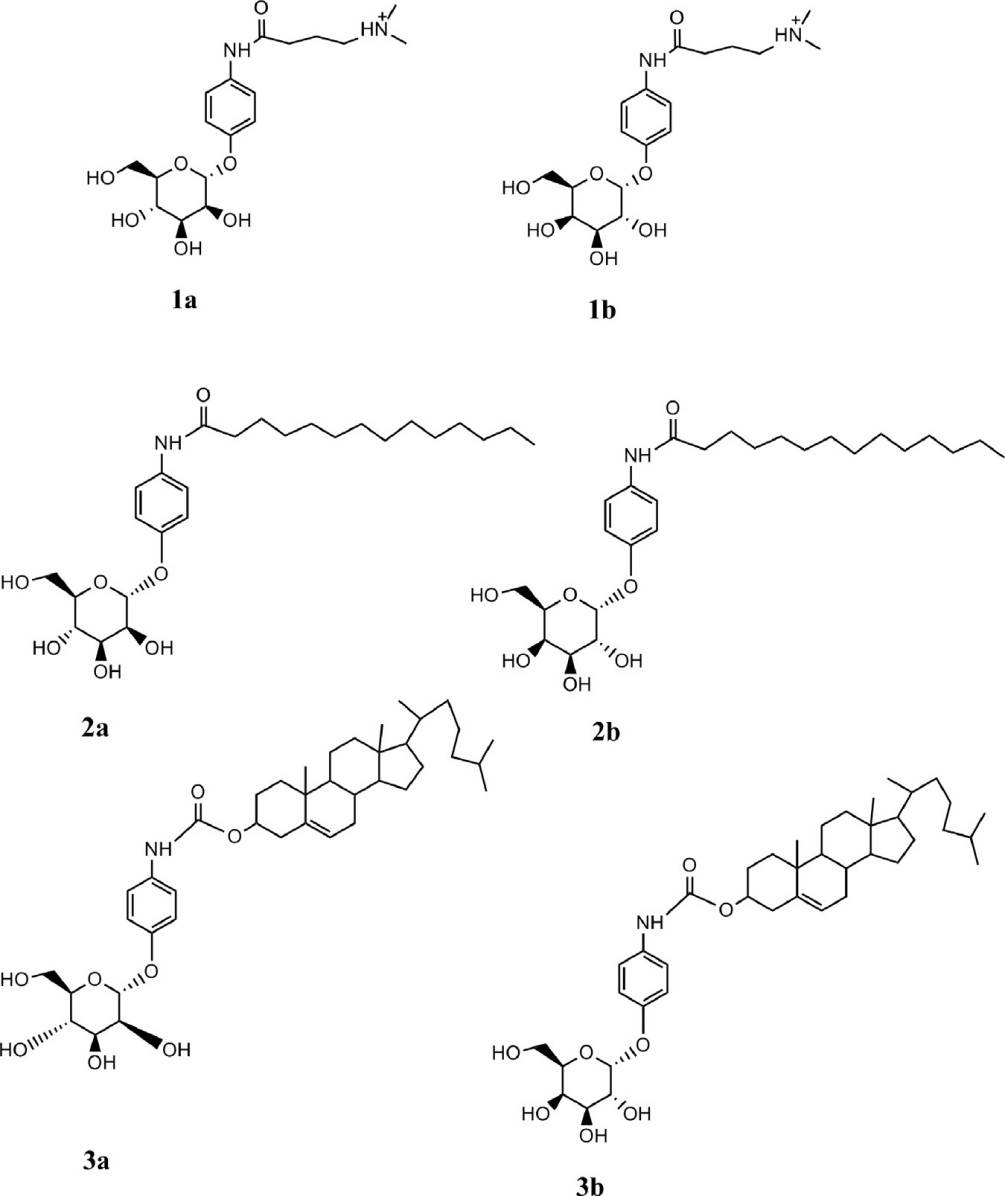
Lipid conjugates of *p*-aminophenyl-α-d-galactopyranoside and *p*-aminophenyl-α-d-mannopyranoside.

**Fig. 2:**
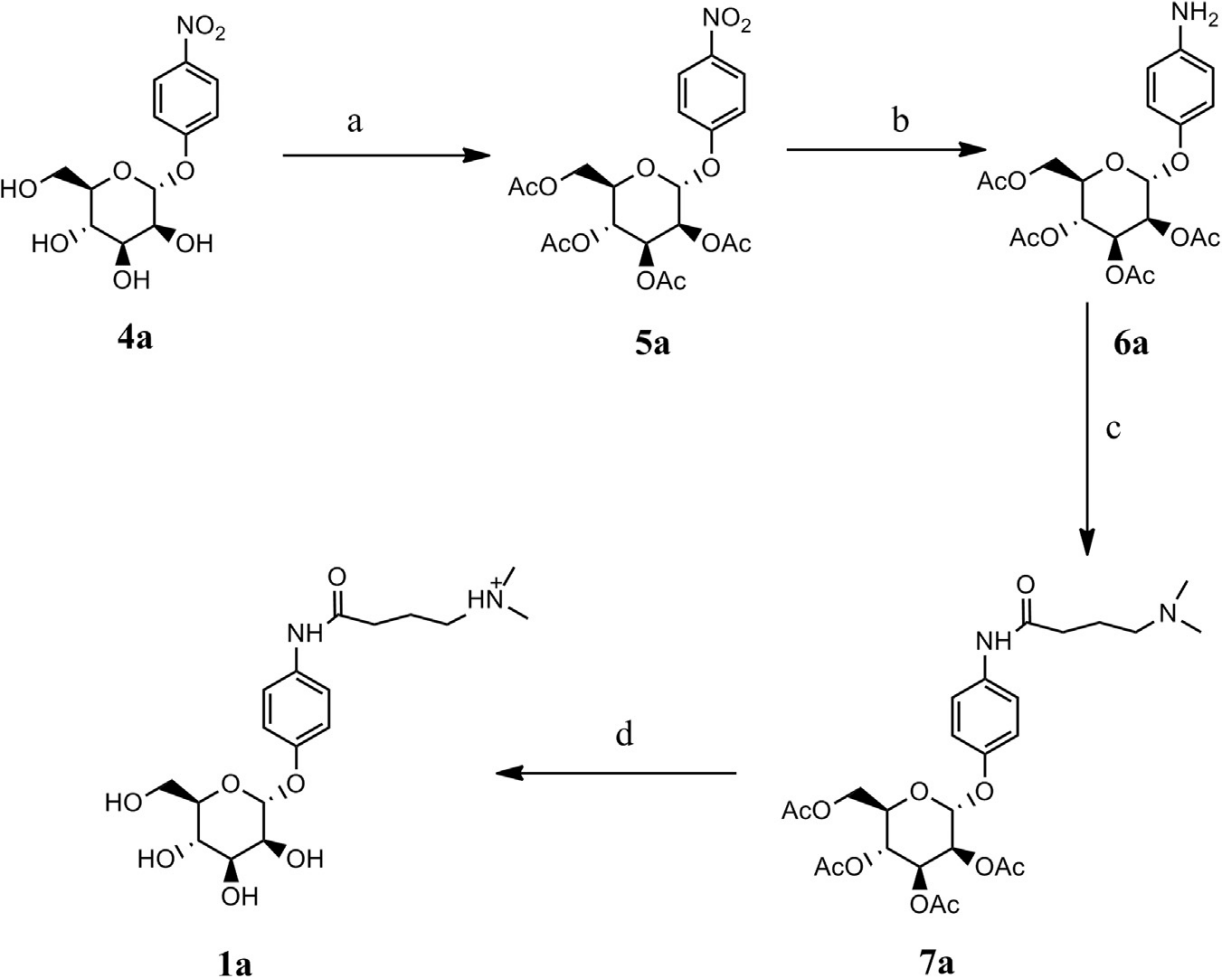
Reagents: (a) Ac_2_O, Pyridine, rt (b) Amm. Formate, Pd-C, MeOH, EtOAc (c) (CH_3_)_2_N(CH_2_)_3_CO_2_H, DCC, DMAP, CH_2_Cl_2_ (d) NaOMe/MeOH.

**Fig. 3:**
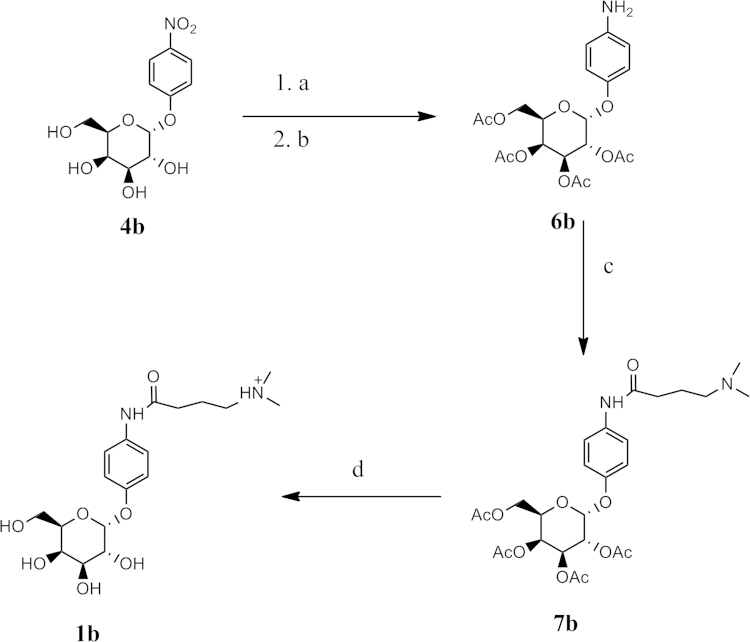
Reagents: (a) Ac_2_O, Pyridine, rt (b) Amm. Formate, Pd-C, MeOH, EtOAc (c) (CH_3_)_2_N(CH_2_)_3_CO_2_H, DCC, DMAP, CH_2_Cl_2_ (d) NaOMe/MeOH.

**Fig. 4:**
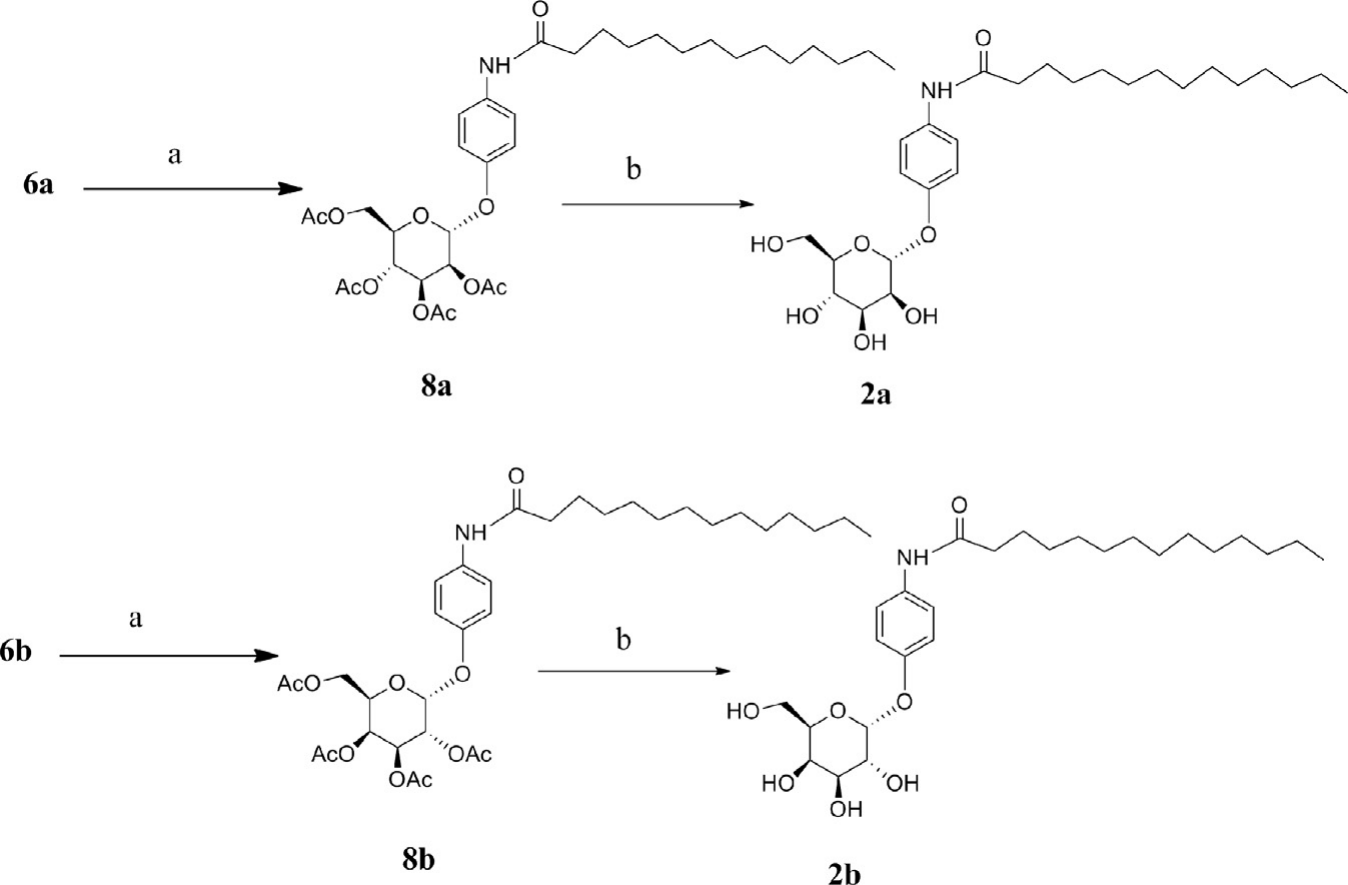
Reagents: (a) Myristic Acid, EDC, DMAP, CH_2_Cl_2_, (b) NaOMe/MeOH.

**Fig. 5:**
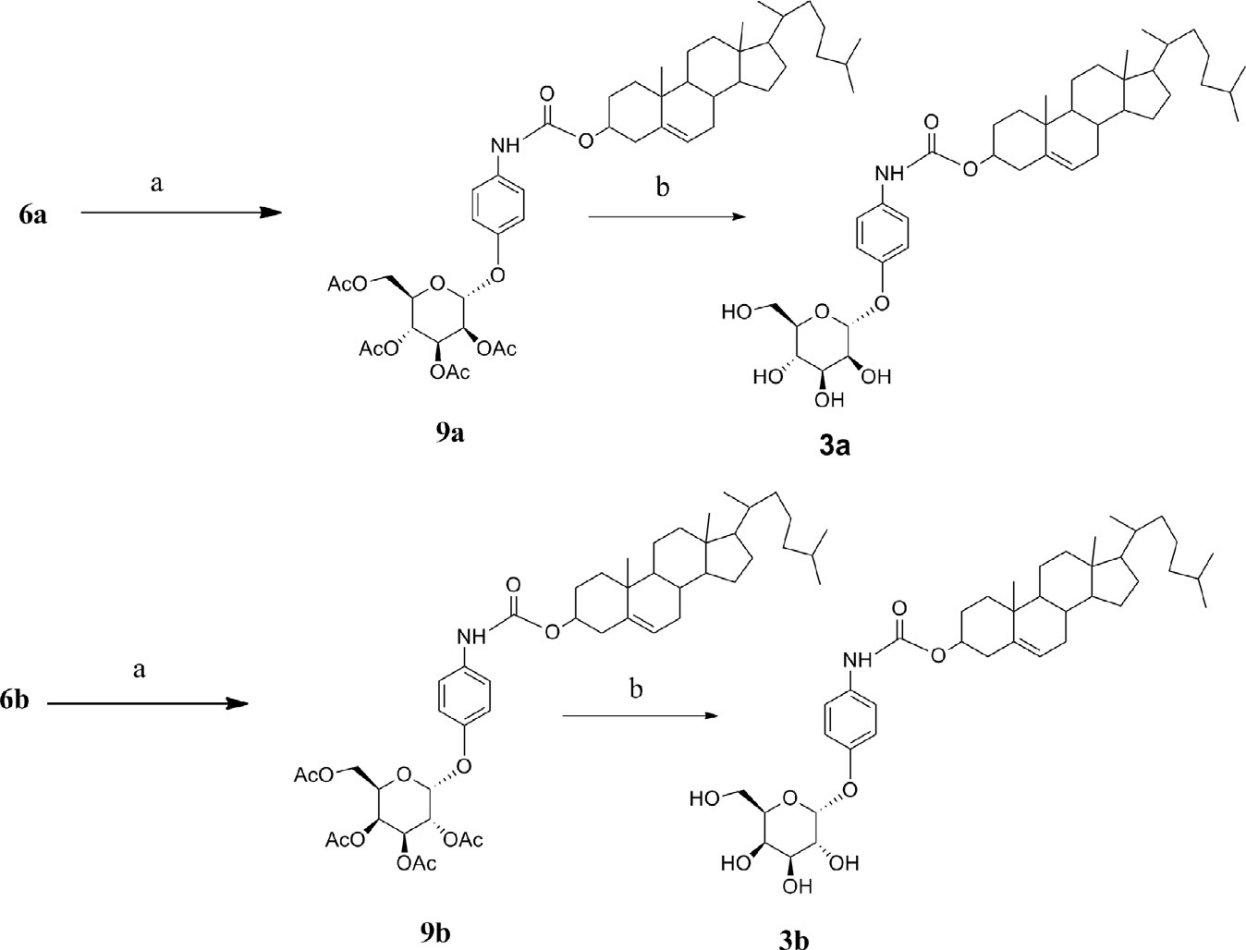
Reagents: (a) Cholesterol Chloroformate, Et_3_N, CH_2_Cl_2_, (b) NaOMe/MeOH.

The schemes for synthesis of carbohydrate-lipid conjugates) are depicted in Figs. 1–5. All the intermediates and final products were characterized by 1H NMR, 13C NMR, IR, HRMS, and optical rotation.

### 1.2 Binding inhibition study

**Fig. 6:**
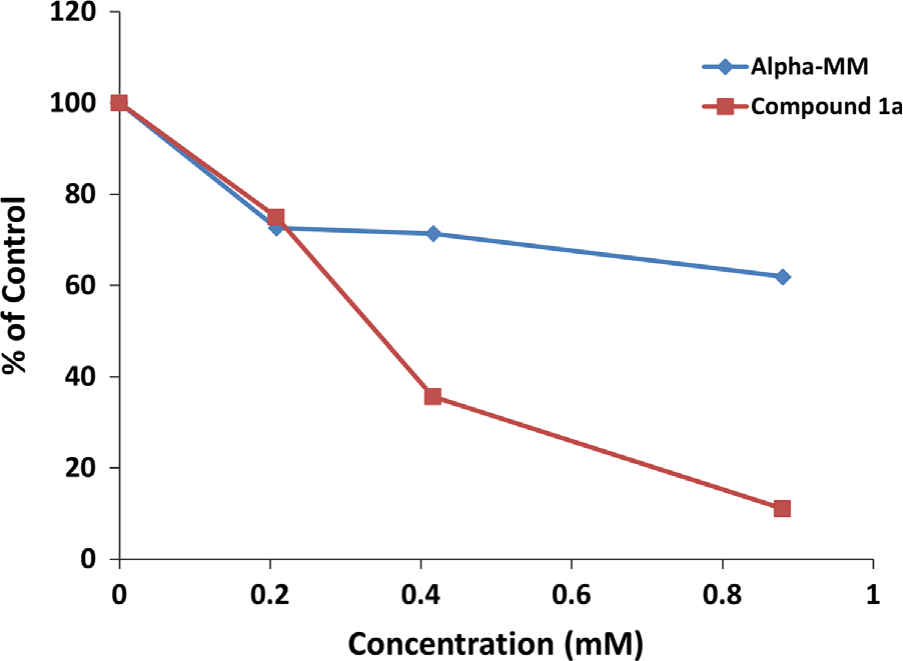
Inhibition of Invertase and Con A aggregation by **1a** and α-MM after incubation at 24.5 °C for 7 min. The inhibition was monitored at OD 320 nm.

**Fig. 7:**
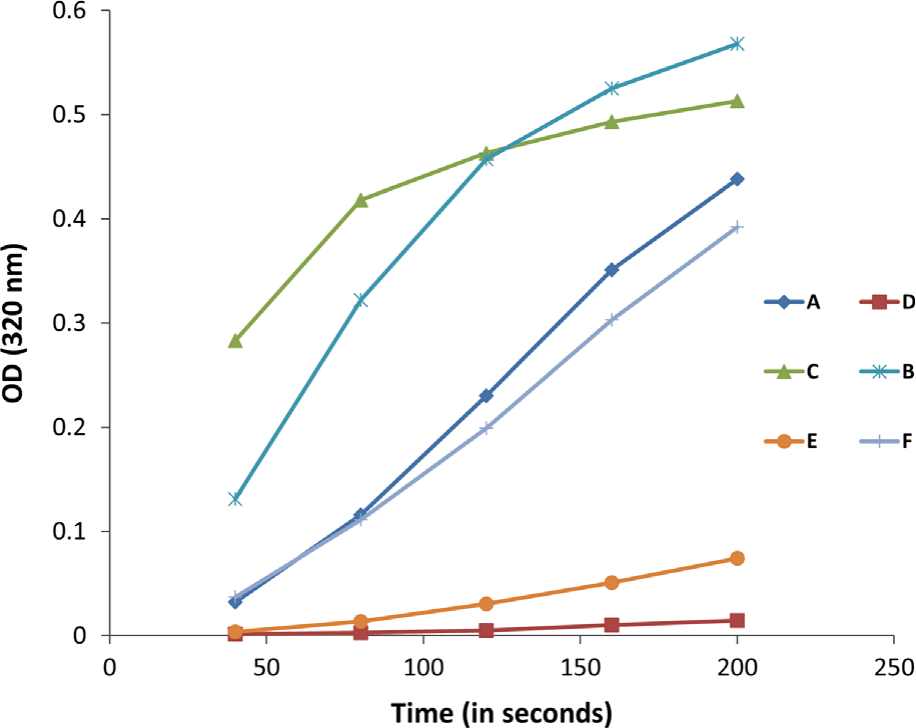
The kinetics of precipitation reaction between Invertase and Con A in the presence of varying amount of α-MM or compound **1a**. (A) α-MM (0.833 mM), Con A and Invertase, (B) α-MM (0.416 mM), Con A and invertase, (C) α-MM (0.208 mM), Con A and invertase, (D) compound **1a** (0.833 mM), con A and invertase, (E) compound **1a** (0.416 mM), Con A and invertase, (F) compound **1a** (0.208 mM), Con A and invertase. The final concentration of Con A and invertase in each test sample were 6.6 µM and 0.66 µM, respectively.

Concanavalin A (Con A), Invertase, and α-MM were purchased from Sigma Aldrich. Solutions of compound **1a** (2.5 mM, 1.25 mM, 0.625 mM), α-MM (2.5 mM, 1.25 mM, 0.625 mM), Con A (20 µM) and Invertase (2 µM) were prepared in phosphate buffered saline (PBS pH 7.4). Solutions of **1a** and α-MM, at various concentrations were mixed with equal volumes of Con A or PBS in triplicate in microtiter plate. Equal volumes of Invertase were added to each well resulting in the final concentration of **1a** and α-MM 0.833 mM, 0.416 mM, and 0.208 mM. The final concentration of Con A, and Invertase in test wells were 6.6 µM and 0.66 µM, respectively. An increase in turbidity at 320 nm (OD320) was monitored at room temperature using SpectraMax M2e (Molecular Devices, CA, USA) (Figs. 6 and 7). Binding of Con A to Invertase was used as control (100%) aggregation and PBS was used a blank.

### 1.3 In vitro transfection

A549 cells (human lung cancer) were obtained from the National Cancer Institute (Frederick, MD) and maintained in Dulbecco’s modified Eagle’s medium (DMEM) supplemented with 10% heat-inactivated fetal bovine serum (FBS). DMEM, FBS and other cell culture reagents were purchased from Life Technologies (Carlsbad, CA).

**Fig. 8:**
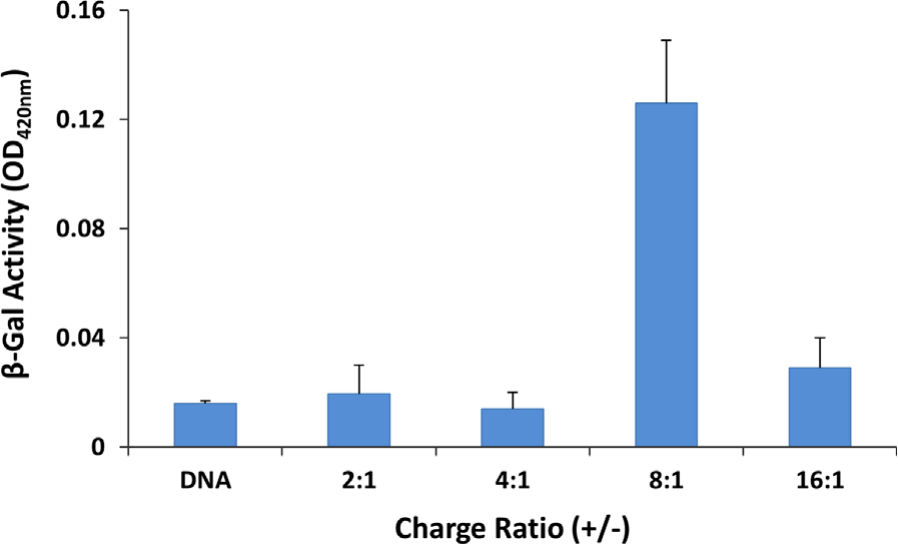
Transfection of pSV-β-gal control vector gene with compound **1a** in A549 cells. Cells were seeded in 96-well plate 24 h before transfection. Cells were incubated with DNA-compound **1a** mixture of different charge ratios for 6 h. The total amount of reporter gene was 0.2 µg/well.

Log-phase A549 cells were seeded in 96-well plates (20,000 cells/well) and cultured overnight in a standard 5% CO_2_ incubator. The culture medium was removed and cells were washed with sterile phosphate-buffered saline (PBS) before adding the transfection mixture. The transfection mixture was prepared by adding reporter gene, pSV-β-gal control vector (Promega, Madison, WI) into equal volume of different concentrations of (**1a**) to yield a series of mixtures with +/‐ charge ratios of 2:1; 4:1; 8:1; 16:1. The mixture was added to and incubated with the cells for 6 h before replaced with regular culture medium. The cells were cultured for a total of 24 h. The total amount of DNA plasmid was 0.2 µg/well in 96-well plates. Both DNA plasmid and **1a** were diluted with OptiMEM I medium (Life Technologies (Carlsbad, CA). The transfection efficiency was determined using β-Galactosidase Enzyme Assay System (Promega, Madison, WI) according to the manufacturer’s protocol (Fig. 8).

